# Autism Treatment Evaluation Checklist (ATEC) Norms: A “Growth Chart” for ATEC Score Changes as a Function of Age

**DOI:** 10.3390/children5020025

**Published:** 2018-02-16

**Authors:** Shreyas Mahapatra, David Vyshedskiy, Samantha Martinez, Benjamin Kannel, Julia Braverman, Stephen M. Edelson, Andrey Vyshedskiy

**Affiliations:** 1 Boston University, One Silber Way, Boston, MA 02215, USA; smahapat@bu.edu (S.M.); sammymartinez9022@gmail.com (S.M.); 2 ImagiRation LLC, 9 Michael Rd, Boston, MA 02135, USA; thedavidv@gmail.com (D.V.); bkannel@fandm.edu (B.K.); braverju@gmail.com (J.B.); 3 Autism Research Institute, 4182 Adams Avenue, San Diego, CA 92116, USA; director@autism.com

**Keywords:** autism, ASD, psychological evaluations, ATEC, Autism Treatment Evaluation Checklist

## Abstract

Most early-intervention Autism Spectrum Disorder (ASD) clinical trials are limited by the availability of psychometric technicians who assess each child’s abilities before and after therapeutic intervention. If parents could administer regular psychometric evaluations of their children, then the cost of clinical trials will be reduced, enabling longer clinical trials with the larger number of participants. The Autism Treatment Evaluation Checklist (ATEC) was designed nearly two decades ago to provide such a tool, but the norms on the longitudinal changes in ATEC in the “treatment as usual” population were lacking. Here we report the norms of the observational cohort who voluntarily completed ATEC evaluations over the period of four years from 2013 to 2017.

## 1. Introduction

The regular assessment of symptom dynamics in children with Autism Spectrum Disorder (ASD) participating in a clinical trial has been a long-standing challenge. A common hurdle in these efforts is the availability of trained technicians needed to conduct rigorous and consistent assessment of children at multiple time points [1,2]. The Autism Treatment Evaluation Checklist (ATEC) was developed to provide a free and easily accessible method for caregivers to track the changes of ASD symptoms over time [3]. Importantly, ATEC was not designed for diagnostic purposes; only to measure changes in ASD severity, making it useful in tracking the efficacy of a treatment. Various studies have sought to confirm the validity of ATEC [4,5,6], yet none to date have assessed typical longitudinal changes in participants’ ATEC scores with respect to age and ASD severity. This paper reports the norms for the longitudinal changes of ATEC scores amongst participants from various countries undergoing a variety of treatments and seeks to develop an outline for tracking developmental changes in individuals with ASD.

ATEC is comprised of four subscales: (1) Speech/Language/Communication, (2) Sociability, (3) Sensory/Cognitive Awareness, and (4) Health/Physical/Behavior. These four subscales are used to calculate a total score that ranges from 0 to 179. A lower score indicates less severe symptoms of ASD and a higher score correlates with more severe symptoms of ASD [3]. The subscales provide survey takers information about specific areas of behavior which may change over time.

Previous studies have aimed to evaluate the validity and reliability of the ATEC in tracking participants’ ASD severity. A trial conducted by Magiati et al., assessed ATEC’s ability to longitudinally measure changes in participant performance [7]. The study utilized ATEC to monitor the progress of 22 schoolchildren over a five-year period. ATEC score was compared to age-specific cognitive, language, and behavior metrics such as the Wechsler Preschool and Primary Scale of Intelligence. The researchers noted ATEC’s high level of internal consistency as well as a highly correlative relationship with other standardized assessments used to measure the same capacities in children with ASD [7]. Charman et al. utilized ATEC amongst other measures in testing the feasibility of caregiver-administered questionnaires to track longitudinal changes in children and noted differential effects across subscales of ATEC in the data collected, possibly driven by development-focused vs. symptom-focused subscales [8]. Another study assessing the ability of dietary intervention to affect ASD symptoms also utilized ATEC as a primary measure [9], concluding that it has “high general reliability” coupled with an ease of access. Whitehouse et al. used ATEC as a primary outcome measure for a randomized controlled trial of their iPad-based intervention for ASD named ‘Therapy Outcomes by You’ (TOBY) [10]. This trial was conducted over a six-month time frame, with outcome assessments at the 3-month and 6-month time points. Although the study did not demonstrate significant ATEC score differences amongst test groups, the researchers reaffirmed their use of ATEC, noting its “internal consistency and adequate predictive validity” [10]. These studies support the viability of ATEC as a tool for longitudinal measurement of ASD severity that can be a vital instrument in tracking symptom changes during a clinical trial.

The current observational study was initiated nearly two decades ago when one of the authors (Dr. Steven Edelson of Autism Research Institute) distributed ATEC questionnaire to parents of children with ASD. Initially, ATEC evaluations were distributed as hard copy. In 2013 the online version of ATEC was developed. The participant responses to the online version of ATEC are presented in this manuscript.

## 2. Methods

### 2.1. The Autism Treatment Evaluation Checklist Structure

The ATEC is a caregiver-administered questionnaire designed to measure changes in severity of ASD in response to treatment. A total score and four subscale scores are reported. Questions in the first three subscales are scored using a 0–2 scale. The fourth subscale, Health/Physical/Behavior, is scored using a 0–3 point scale. ATEC can be accessed online or in hard-copy format.

The first subscale, Speech/Language/Communication, contains 14 items where the score ranges from 0–28 points. The Sociability subscale contains 20 items and participants can score from 0–40. The third subscale, Sensory/Cognitive awareness, has 18 items and scores range from 0–36. Finally, the Health/Physical/Behavior subscale contains 25 items. The scores from each subscale are combined in order to calculate a Total Score, which ranges 0–179 points. A lower score indicates a lower severity of ASD symptoms.

### 2.2. Collection of Evaluations

ATEC responses originated from participants voluntarily and freely completing ATEC evaluations online from 2013 to 2017. Using the Department of Health and Human Services regulations found at 45 CFR 46.101(b)(4), the Chesapeake Institutional Review Board (IRB) determined that this research project is exempt from IRB oversight.

### 2.3. Calculations of the ATEC Norms

**Figure 1:**
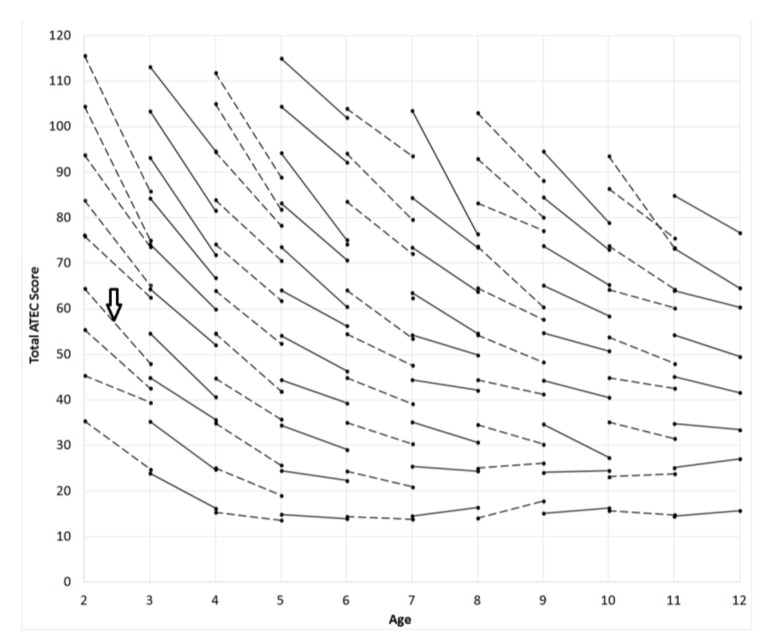
Pair-wise changes in the total Autism Treatment Evaluation Checklist (ATEC) score from one whole-year age to another. Each point represents an average of all participants who completed their first evaluation at one whole-year age and their second evaluation at a following whole-year age. For example, in the 2 to 3 age-pair calculation norms (vertical arrow), a participant must have completed their first evaluation anytime between the ages 1.5 to 2.5 years and a second evaluation between the ages 2.5 and 3.5 years.

In order to generate ATEC norms, changes in score from one whole-year age to another were calculated for each participant (Figure 1). For these calculations, participants who had completed at least one evaluation at two consecutive year-age time-points were selected. For example, in the 2 to 3 age-pair norms calculation, a participant must have completed their first evaluation anytime between the ages 1.5 to 2.5 years and a second evaluation between the ages 2.5 and 3.5 years. When more than one evaluation was completed by a participant at any given age, the evaluations were averaged. Thus, for each age-year time-point, a pair of values was generated that characterized a participant’s ATEC score change from one chronological year to another. Note that most participants did not complete ATEC over multiple years and thus provided only a single pair of data points. Participants were then sorted by their initial ATEC total score into bins in 10-point increments. The lines in Figure 1 connect the average scores in each pair of these observations.

**Figure 2:**
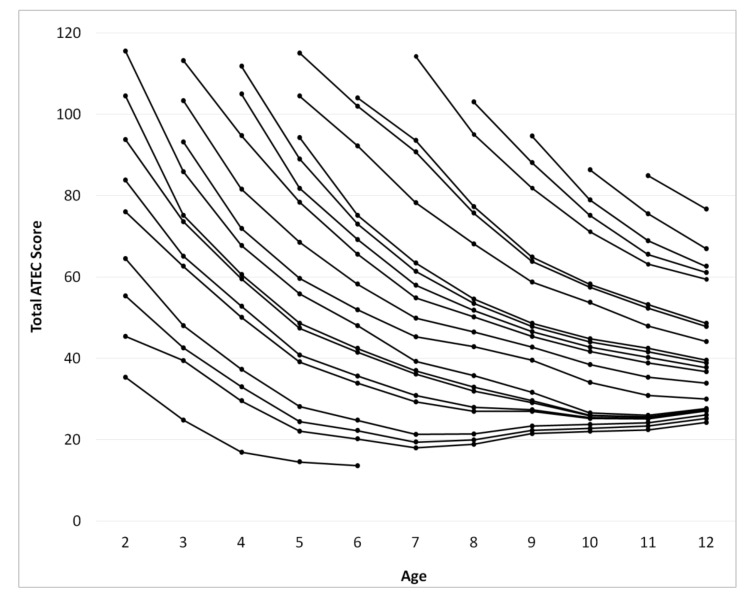
Continuous changes of the total ATEC score over age calculated from pair-wise observations using the “relay” procedure. To find out a typical trajectory for a child, (1) find the point on the graph corresponding to the child’s current age and score; (2) locate the trajectory line under the point; (3) follow the trajectory line to read the typical ATEC total score at any age. For example, a typical child with ATEC total score of 116 at age 2 is expected to reduce ATEC total score to 86 at age 3; 68 at age 4; 56 at age 5; 48 at age 6; 39 at age 7; 36 at age 8; 32 at age 9; 27 at age 10; 26 at age 11; and 28 at age 12.

From the pair-wise observations generated in the process detailed above, a measure of the continuous changes in ATEC score over the participants’ age was developed to define norms. In order to convert the pair-wise ATEC score lines shown in Figure 1 to continuous distributions, inferences about ATEC score changes between age bins were made. For example, participants with an initial ATEC total score of 64 at the age of 2 years had on average reduced their score to 48 by the age of 3 years (Figure 1, vertical arrow). The change in the score from the age of 3 years to the age of 4 years in participants with the average score of 48 at the age of 3 years, however, is unknown. To mitigate this uncertainty, a statistical inference was made utilizing the two numerically closest observations at a given age. In this example, the two closest defined values to the score of 48 at the age of 3 years are 55 and 45. The participants with initial score of 55 at the age of 3 years have on average reduced their score to 41 by the age of 4 years. Those participants with initial score of 45 at the age of 3 years have on average reduced their score to 36 by the age of 4 years. The absolute distance at the age of 3 years (55 – 48 = 7 and 48 – 45 = 3) was used to normalize the inferred score of 37 at the age of 4 years. Thus, a corresponding ATEC total score value at the age of 4 years was generated to link the score 48 at the age of 3 years to the inferred score of 37 at the age of 4. This “relay” procedure was used to approximate the score at the ages from 3 to 12 years, for all bins that contained the data from 5 or more participants, Figure 2.

### 2.4. Participants

Participants were selected based on the following criteria:

1. Completeness: Participants who did not provide a date of birth (DOB) were excluded. As participants’ DOB were utilized to determine age, the availability of DOB was a vital factor.
2. Consistency: Participants must have filled out at least three questionnaires and the interval between the first and the last evaluation was one year or longer.
3. Maximum age: Participants older than 12.5 years of age were excluded from this study.
4. Minimal ATEC severity: Participants with average ATEC total scores less than 20 were excluded.

After excluding participants that did not meet the aforementioned criteria for the study, there were 2649 total participants. Among the 2649 participants there were 2187 males (83%) and 444 females (17%) (18 individuals did not specify their sex).

## 3. Results

Utilizing the “relay” procedure, changes in ATEC total score as a function of age were calculated from pair-wise observations in 2649 participants (Figure 2). A decrease in ATEC total score indicates an improvement of participants’ symptoms. Mathematically, the decrease of the ATEC total score is best described by an exponent with a time constant of 3.3 years decaying to a constant baseline. The constant baseline scores were proportional to ATEC total score at the age of 2 years, indicating predictive power of this score for ASD symptom severity later in life. This relationship between the ATEC total score and age for different initial values of ATEC total score are presented in Table 1.

**Figure 3:**
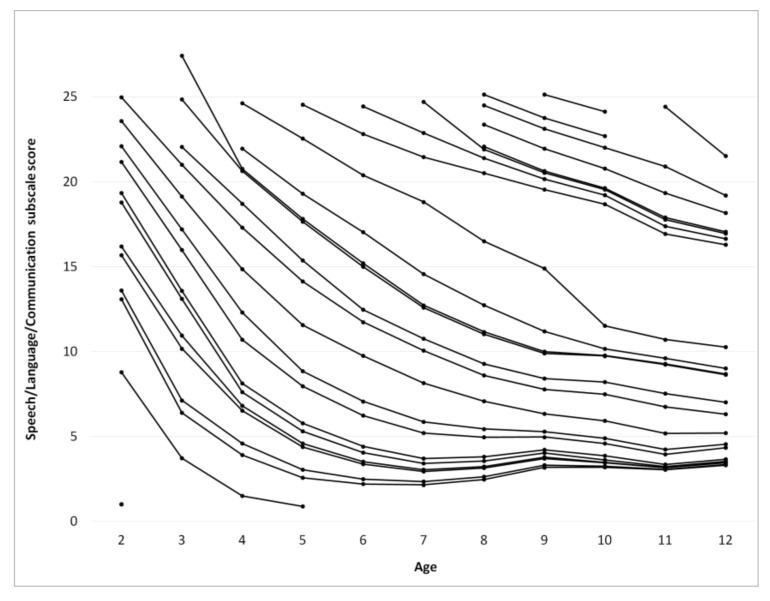
Continuous changes of the Speech/Language/Communication subscale score over age calculated from pair-wise observations using the “relay” procedure.

**Figure 4:**
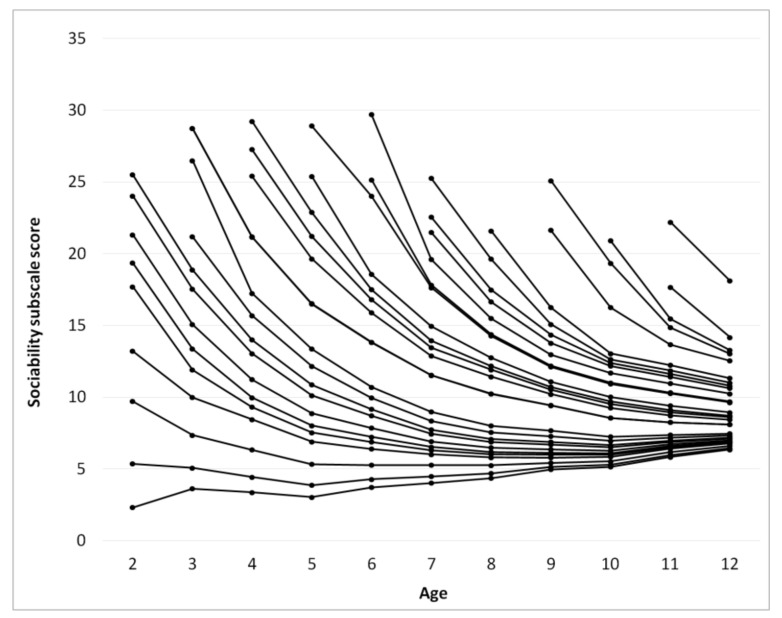
Continuous changes of the Sociability subscale score over age calculated from pair-wise observations using the “relay” procedure.

**Figure 5:**
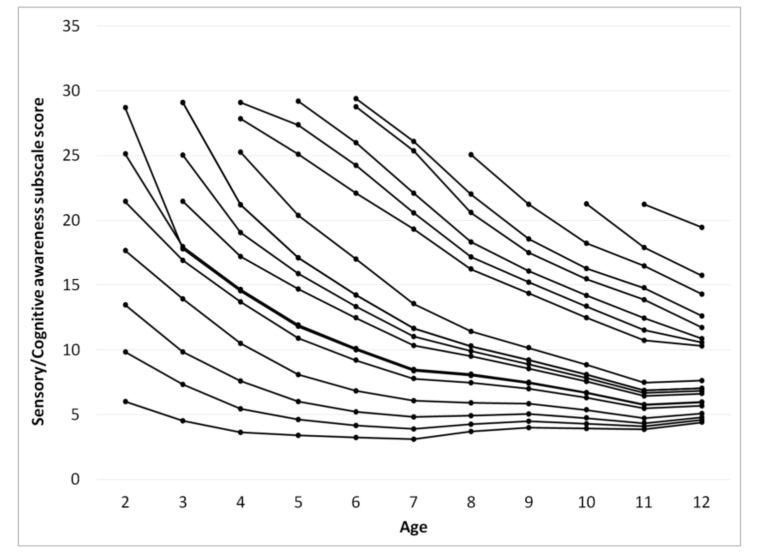
Continuous changes of the Sensory/Cognitive awareness subscale score over age calculated from pair-wise observations using the “relay” procedure.

**Figure 6:**
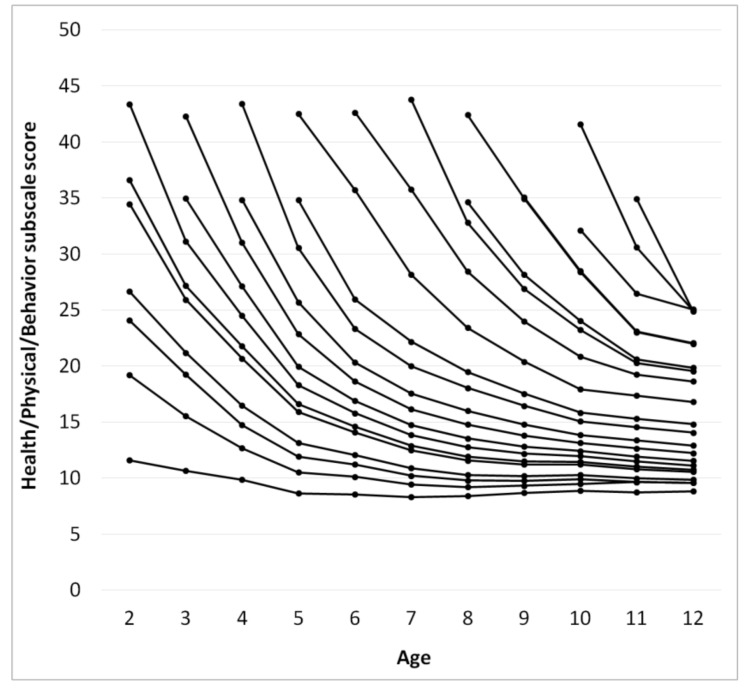
Continuous changes of the Health/Physical/Behavior subscale score over age calculated from pair-wise observations using the “relay” procedure.

The score for each subscale as a function of age was calculated using a procedure identical to that used for calculating ATEC total score, Figure 3, Figure 4, Figure 5 and Figure 6. Table 2, Table 3, Table 4 and Table 5 present the developmental norms for each subscale.

## 4. Discussion

Design considerations for an early-intervention clinical trial for ASD must take into account (1) the trial duration, (2) number of participants, and (3) the quality of participant assessment. A short clinical trial of an early therapeutic intervention in two- to three-year-old children can easily miss a target, as an improvement of symptoms may not emerge until children reach the school age. Small numbers of participants can easily skew the data as ASD is known to be a highly heterogeneous disorder. Trial duration and numbers of participants both serve as key measures of the rigor of a clinical trial for any therapeutic intervention. Increasing the clinical trial duration and the number of trial participants, however, raises the demand for regular assessment of participants by trained psychometric technicians. Furthermore, to attain a larger number of trial participants, clinical trials must accept participants across a large geographical region. The logistical issues associated with such an endeavor come at immense cost. As a result, large numbers of ASD clinical trials working under a limited budget suffer from short duration and low participant number, often compromising the trial objectives (e.g., [10,11]).

ATEC was in part designed to circumvent these problems. If caregivers could serve as psychometric technicians and conduct regular evaluations of their children, the cost of clinical trials will be substantially reduced while simultaneously allowing for longer trial duration. This manuscript attempts to characterize the typical changes in ATEC score over time as a function of children initial age and ASD severity in a large and diverse group of participants. In doing so, it lends support to the efficacy of caregiver-driven psychometric observation, which when applied at scale, may be a viable alternative to using licensed technicians to assess the children.

### 4.1. Utility of Norms for Tracking Developmental Trajectory

A primary goal in developing the continuous distribution charts in this paper is to provide a basis for tracking development in individuals with ASD. Currently no easily accessible childhood metric for ASD development exists. The “gold standard” for observational assessment of an ASD is the Autism Diagnostic Observation Schedule (ADOS) [12]. The Childhood Autism Rating Scale (CARS) [13] and Mullen Scales of Early Learning (MSEL) [14] are also widely used as ASD assessment tools. All of these tools are expensive instruments, designed to be administered by a trained examiner, and not readily available to caregivers. Furthermore, none of these tools have published developmental norms [15].

In utilizing ATEC norms described in this manuscript, caregivers will be able to evaluate their child’s ASD severity at any age and also track its trajectory and project future ASD severity. These distributions serve to establish norms for ASD trajectory and are intended to be utilized by caregivers to identify and track a subject’s severity across development. When tracked annually, these distributions may function much like childhood growth charts utilized by physicians to track childhood physical development. Caregivers will also be able to engage in informed discussion with therapists with regard to therapy effectiveness.

### 4.2. Changes in Autism Treatment Evaluation Checklist Total Score and Subscale Scores

In modeling the continuous changes in ATEC total score (Figure 2) across a participant’s development, interesting qualitative observations can be made. Regardless of initial severity, participants’ ATEC total scores display exponential decrease with a time constant of approximately 3.3 years. A similar trend is observed in all subscales and may indicate normal developmental changes. Participants exhibiting an ATEC total score above 70 at the age of two years improve their symptoms exponentially but seem to reach a constant baseline around the age of 12. The score at the baseline is proportional to the Total score at the age of two. In other words, the ATEC total score at the age of 12 may be predicted from the total score at the age of two years.

Surprisingly, for participants with an ATEC total score below 70 at the age of two years the ATEC total score increases after the age of 7 indicating deterioration of symptoms. This increase in the score is observed in the Communication subscale (Figure 3), the Sociability subscale (Figure 4), and the Sensory subscale (Figure 5), but is absent in the Physical subscale (Figure 6). This deterioration of symptoms may be attributed to different interpretation of ATEC questions at different ages. Consider the Sociability subscale, which shows most significant deterioration of symptoms (Figure 4). Question 11: “Dislikes being held/cuddled” can clearly be interpreted differently at age 2 and age 7: a parent can nearly always cuddle a two-year-old, but not a seven-year-old. Other symptoms described in the Sociability subscale, such as questions 6: “Prefers to be left alone”, 12: “Does not share or show”, 16: “Lacks friends/companions”, may not be relevant to a toddler at all, resulting in a parent answering “not descriptive.” At the age of seven, however, the same parent may decide that these symptoms accurately describe their child and therefore alter their responses, increasing the Sociability subscale score as well as ATEC total score.

## 5. Limitations

Participant selection presents a novel challenge in a study focused on caregiver-administered assessments. In the selection of participants for inclusion in this study, a baseline of ASD diagnosis could not be established as child’s diagnosis is not part of ATEC questionnaire. Thus, some of the participants may have been lacking ASD diagnosis altogether. For example, parents of a neurotypical toddler worried for any reason about an ASD diagnosis could have decided to monitor toddler’s development with ATEC evaluations and thus inadvertently added their normally developing child to the ATEC collection. As neurotypical children develop faster, the presence of neurotypical children in the dataset would have artificially increased the magnitude of annual changes of ATEC scores, predominantly for younger participants with mild ASD.

A requirement for ASD diagnosis, however, would have presented its own set of challenges. Notably, as ASD diagnoses are not apparent for many years, any potential data that could have been gathered from younger individuals would need to be eliminated until the confirmation of the diagnosis. This issue is compounded by diagnostic recommendations that are geographically inconsistent, resulting in variable selection criteria.

For multiple reasons, it is unlikely that there were many neurotypical participants in the database utilized for this paper. First, the ATEC questionnaire is virtually unknown outside the autism community. Second, there is little incentive for the parents of neurotypical children to complete multiple exhaustive ATEC questionnaires (unless one of the children was previously diagnosed with ASD). Third, to further limit the contribution from neurotypical children, we excluded participants with an average ATEC total score of 20 or less, that may have represented the neurotypical population (7% of all participants). Despite this effort, the reported data may over-approximate the magnitude of annual changes of ATEC scores, especially in the younger participants with mild ASD.

Another limitation is associated with the wide definition of a whole-year age established to assess pair-wise changes in the ATEC score from one age to another. For example, it is possible that a parent(s) administered the checklist at 2.4 years of age and then 2.6 years of age and therefore have been assigned to two different whole-year ages while another parent(s) administered the checklist at 1.6 and 3.4 years of age; both parents would have been assigned to the same whole-year age pair: 2 and 3. Further studies with larger number of participants should be able to shorten the age group definition from whole-year to six months and possibly even three months.

There is an understanding in the psychology community that parents cannot be trusted with an evaluation of their own children. In fact, parents often yield to wishful thinking and overestimate their children’s abilities on a single assessment. However, by measuring the change in score over multiple assessments, pattern of changes could be extracted. When a single parent completes the same evaluation every three months over multiple years, changes in the score become meaningful.

As noted previously by other groups [10,8], the use of ATEC as a primary outcome measure has some inherent drawbacks. While the ATEC is capable of delineating incremental differences in ASD severity amongst participants, the variety of measures amongst its subscales fails to differentiate developmental-specific from symptom-specific changes. This aspect of the ATEC may introduce a confounding variable when participants are at different developmental stages and on unique developmental trajectories during a study. As noted previously, certain phenomena observed in ATEC score changes may be an artifact of different caregiver interpretations of behaviors at different ages. To mitigate these effects, trial designs must accurately separate participants based on developmental stage. This is most often accomplished by using age as a proxy for developmental stage.

Another limitation of this study is lack of stratification based on presence/absence of diagnosis, age of diagnosis, lack of data analysis by sex, race, socio-economic status or presenting syndrome. Unfortunately, data on presence/absence of diagnosis, age of diagnosis, race, socio-economic status and presenting syndrome were not collected. The data on participant’s sex were collected and a linear mixed effects model was used to evaluate longitudinal changes in ATEC scores. The model showed no difference in improvement between the two sex groups [16]. One surprising finding was that children from developed English-speaking countries improved less than children from the rest of the world [16]. Accordingly, an attempt was made to generate norms separately for developed English-speaking countries and the rest of the world. The reduction of the number of participants in each group resulted in shorter intermittent normative trajectory lines and, therefore, was rejected by the authors. Future studies with greater number of participants shall generate the normative data separately for groups with statistically different longitudinal changes.

## 6. Conclusions

A primary purpose of this study was to generate developmental norms for the ATEC to be used as an outcome measure for longitudinal assessment of individuals with ASD. Parents, therapists and researchers may use these norms to compare the development of a child with ASD to a large group of individuals with the same diagnosis.

## Figures and Tables

**Table 1:** The Total ATEC score norms as a function of the initial score and age from Figure 2. To find out a typical score trajectory for a child, (1) find the column with the child’s current age; (2) find the line with the child’s current ATEC total score; (3) in the same line, read typical ATEC total score at any age. For example, a typical child with ATEC total score of 116 at age 2 is expected to reduce ATEC total score to 86 at age 3; 68 at age 4; 56 at age 5; 48 at age 6; 39 at age 7; 36 at age 8; 32 at age 9; 27 at age 10; 26 at age 11; and 28 at age 12.

Age										
2	3	4	5	6	7	8	9	10	11	12
35	25	17	15	14						
45	40	30	22	20	18	19	21	22	22	24
55	43	33	24	22	19	20	22	23	23	25
64	48	37	28	25	21	21	23	24	24	26
76	63	50	39	34	29	27	27	25	25	27
84	65	53	41	36	31	28	27	25	25	27
94	74	60	47	42	36	32	29	26	26	27
105	75	61	49	42	37	33	30	26	26	27
116	86	68	56	48	39	36	32	27	26	28
	93	72	60	52	45	43	40	34	31	30
	103	82	69	58	50	46	43	39	35	34
	113	95	78	66	55	50	45	42	39	37
		105	82	69	58	52	47	43	40	38
		112	89	73	61	54	48	44	42	39
			94	75	63	55	49	45	42	40
			104	92	78	68	59	54	48	44
			115	102	91	76	64	58	52	48
				104	94	77	65	58	53	49
					114	95	82	71	63	59
						103	88	75	66	61
							95	79	69	63
								86	76	67

**Table 2:** Speech/Language/Communication subscale score norms as a function of the initial score and age from Figure 3.

Age										
2	3	4	5	6	7	8	9	10	11	12
9	4	2	1							
13	6	4	3	2	2	2	3	3	3	3
14	7	5	3	2	2	3	3	3	3	3
16	10	7	4	3	3	3	4	3	3	3
16	11	7	5	4	3	3	4	3	3	3
19	13	8	5	4	3	4	4	4	3	4
19	14	8	6	4	4	4	4	4	3	4
21	16	11	8	6	5	5	5	5	4	4
22	17	12	9	7	6	5	5	5	4	5
24	19	15	12	10	8	7	6	6	5	5
25	21	17	14	12	10	9	8	7	7	6
	22	19	15	12	11	9	8	8	8	7
	25	21	18	15	13	11	10	10	9	9
	27	21	18	15	13	11	10	10	9	9
		22	19	17	15	13	11	10	10	9
		25	23	20	19	16	15	12	11	10
			25	23	21	20	20	19	17	16
				24	23	21	20	19	17	17
					25	22	21	20	18	17
						22	21	20	18	17
						23	22	21	19	18
						24	23	22	21	19
						25	24	23		
							25	24		
									24	22

**Table 3:** Sociability subscale score norms as a function of the initial score and age from Figure 4.

Age										
2	3	4	5	6	7	8	9	10	11	12
2	4	3	3	4	4	4	5	5	6	6
5	5	4	4	4	4	5	5	5	6	6
10	7	6	5	5	5	5	5	6	6	7
13	10	8	7	6	6	6	6	6	6	7
18	12	9	8	7	6	6	6	6	7	7
19	13	10	8	7	7	6	6	6	7	7
21	15	11	9	8	7	6	6	6	7	7
24	18	13	10	9	7	7	7	7	7	7
26	19	14	11	9	8	7	7	7	7	7
	21	16	12	10	8	8	7	7	7	7
	26	17	13	11	9	8	8	7	7	7
	29	21	17	14	12	10	9	9	8	8
	29	21	17	14	12	10	9	9	8	8
		25	20	16	13	11	10	9	9	8
		27	21	17	13	12	11	10	9	9
		29	23	18	14	12	11	10	9	9
			25	19	15	13	11	10	9	9
			29	24	18	14	12	11	10	10
				25	18	14	12	11	10	10
				30	20	15	13	12	11	10
					21	17	14	12	11	11
					23	17	14	12	12	11
					25	20	15	13	12	11
						22	16	13	12	11
							22	16	14	13
							25	19	15	13
								21	15	13
									18	14
									22	18
										

**Table 4:** Sensory/Cognitive awareness subscale score norms as a function of the initial score and age from Figure 5.

Age										
2	3	4	5	6	7	8	9	10	11	12
6	5	4	3	3	3	4	4	4	4	4
10	7	5	5	4	4	4	4	4	4	5
13	10	8	6	5	5	5	5	5	4	5
18	14	10	8	7	6	6	6	5	5	5
21	17	14	11	9	8	7	7	6	5	6
29	18	15	12	10	8	8	7	7	6	6
25	18	15	12	10	8	8	8	7	6	6
	21	17	15	12	10	10	9	8	6	7
	25	19	16	13	11	10	9	8	7	7
	29	21	17	14	12	10	9	8	7	7
	29	21	17	14	12	10	9	8	7	7
		25	20	17	14	11	10	9	7	8
		28	25	22	19	16	14	12	11	10
		29	27	24	21	17	15	13	12	11
			29	26	22	18	16	14	12	11
				29	25	21	17	15	14	12
				29	26	22	19	16	15	13
						25	21	18	16	14
								21	18	16
									21	19

**Table 5:** Health/Physical/Behavior subscale score norms as a function of the initial score and age from Figure 6.

Age										
2	3	4	5	6	7	8	9	10	11	12
12	11	10	9	9	8	8	9	9	9	9
19	16	13	11	10	9	9	9	9	10	10
24	19	15	12	11	10	10	10	10	10	10
27	21	16	13	12	11	10	10	10	10	10
34	26	21	16	14	12	12	11	11	11	11
37	27	22	17	15	13	12	11	11	11	11
43	31	24	18	16	14	13	12	12	11	11
	35	27	20	17	15	14	13	12	12	12
	42	31	23	19	16	15	14	13	13	12
		35	26	20	18	16	15	14	13	13
		43	31	23	20	18	16	15	15	14
			35	26	22	19	18	16	15	15
			43	36	28	23	20	18	17	17
				43	36	28	24	21	19	19
					44	33	27	23	20	20
						35	28	24	21	20
						42	35	28	23	22
							35	28	23	22
								32	26	25
								42	31	25
									35	25
